# Immigrant Mental Health, A Public Health Issue: Looking Back and Moving Forward

**DOI:** 10.3390/ijerph121013624

**Published:** 2015-10-27

**Authors:** Usha George, Mary S. Thomson, Ferzana Chaze, Sepali Guruge

**Affiliations:** 1 Faculty of Community Services, Ryerson University, 99 Gerrard Street East, SHE-690; 350 Victoria Street, Toronto, ON M5B 2K3, Canada; E-Mail: marysusan.thomson@ryerson.ca; 2 Community Studies, Sheridan College, 7899 McLaughlin Road, Brampton, ON L6Y 5H9, Canada; E-Mail: ferzana.chaze@sheridancollege.ca; 3 School of Nursing; Ryerson University, Faculty of Community Services; 350 Victoria Street, Toronto, ON M5B 2K3, Canada; E-Mail: sguruge@ryerson.ca

**Keywords:** immigrants, settlement, mental health, public health, Canada

## Abstract

The Mental Health Commission of Canada’s (MHCC) strategy calls for promoting the health and wellbeing of all Canadians and to improve mental health outcomes. Each year, one in every five Canadians experiences one or more mental health problems, creating a significant cost to the health system. Mental health is pivotal to holistic health and wellbeing. This paper presents the key findings of a comprehensive literature review of Canadian research on the relationship between settlement experiences and the mental health and well-being of immigrants and refugees. A scoping review was conducted following a framework provided by Arskey and O’Malley (Int J Soc Res Methodol 8:19–32, 2005). Over two decades of relevant literature on immigrants’ health in Canada was searched. These included English language peer-reviewed publications from relevant online databases Medline, Embase, PsycInfo, Healthstar, ERIC and CINAHL between 1990 and 2015. The findings revealed three important ways in which settlement affects the mental health of immigrants and refugees: through acculturation related stressors, economic uncertainty and ethnic discrimination. The recommendations for public health practice and policy are discussed.

## 1. Introduction

Public health is concerned with the prevention of disease and with the promotion and protection of health in ways that promote social justice [1]. Mental health is central to health [2]. World Health Organization’s (WHO) concept of mental health includes the promotion of mental well-being, the prevention and treatment of mental illness, as well as the rehabilitation of persons affected by mental illness ([2,3], p. 1). Research has recognized the vulnerability of immigrants and refugees in relation to mental health [4,5,6,7,8,9,10,11,12]. In 2011 over 20% of the total Canadian population was foreign-born. Over 17% of the foreign-born population were recent immigrants between 2006 and 2011 [13]. This paper presents the findings of a scoping review that focuses on the relationship between settlement experiences and mental health and wellbeing for immigrants and refugees in Canada. It builds on existing knowledge in capturing the mental health needs of diverse immigrant groups and makes a case for a holistic approach to public health intervention with immigrants and refugees. The mental health needs and importance of public health intervention for many small communities are not well captured in most studies using national samples.

In this review we are trying to narrow this gap in the literature. The paper is divided into four sections. Following this introduction we provide a brief background that helps us contextualise the impact of settlement experiences on immigrants’ mental health. The second section details the methods used in the review. Section three discusses the findings of the review and section four discusses the recommendations for public health that emerge from the review.

### Background

The process of adapting to the host country can be a stressful process, requiring psychological and socio-cultural adaptations [14]. Since the 1990s Canada has been accepting over 200,000 immigrants from around the world [15]. In the past few decades, these immigrants have typically migrated from countries in Asia and Africa. Coming from diverse cultures these racialized immigrants are likely to experience psychological stressors in the process of acculturation what Berry terms “acculturative stress” ([14], p. 9).

A majority of immigrants to Canada are “economic immigrants”, accepted on the basis of their potential to contribute to the Canadian labor market through a points system based on language proficiency, professional qualifications and work experience. Research has demonstrated that though a majority of immigrants to Canada are carefully selected on basis of such a merit based point system, many are unable to find work commensurate with their education and training [16,17,18]. Despite having higher educational qualifications compared to native born persons, immigrants are more likely to be underemployed compared to native born Canadians [19,20].

Immigrants face many barriers in accessing employment in Canada such as the lack of acceptance of their foreign credentials by professional bodies and employers [21,22,23], language related barriers including discrimination on account of speaking English with a foreign accent [24,25,26,27,28], and lack of prior Canadian work experience [28,29,30]. The inability to secure suitable work forces many immigrants to take up low skilled, precarious work to survive [19,31]. Further, immigrants earn less on the job compared to native born persons doing similar work [32]. It is not surprising then, that immigrants are one of the five main poverty affected groups in Canada [33,34].

Though new immigrants to the country have better health than their native-born counterparts a phenomenon termed the “healthy immigrant effect” [35], their health advantage decreases over the years in the country [35,36]. The phenomenon is indicative of the negative impacts of migration on immigrant health [36].

That the health and mental health of an individual is influenced by many social factors is well established [3,37]. Migration has been acknowledged as one of the SDH [38] as migrants may face poverty, social isolation, and social inequities in their host countries. The Public Health Agency of Canada notes many factors as determinants of health including income, social networks, employment and having a minority culture and associated risk of marginalization [37]. As we have seen immigrants are vulnerable to many of these determinants.

## 2. Experimental Section

### Methodology

This paper presents the findings of a scoping review that focuses on the relationship between settlement experiences and mental health for immigrants in Canada. Arskey and O’Malley [39] define scoping reviews as reviews that “aim to map rapidly the key concepts underpinning a research area and the main sources and types of evidence available” (p. 5). This methodology was deemed as most appropriate because of its systematic nature and its use in several other scoping reviews to map out a large area of research and explore issues in health among immigrants and refugees. Our objectives for conducting this review aligned with Arskey and O’Malley’s objectives (i) to examine the extent, range and nature of research activity (ii) to summarize and disseminate research findings and (iii) to identify research gaps in literature. Our aim and broader research question was to map the key findings from published literature in our fields of interest *i.e.*, immigrant and refugee health in Canada, health determinants including pre and post migration experiences and identify the gaps in this area of literature. We did not differentiate between immigrants and refugees for the purpose of this review. The stages involved in the review process, guided Arskey and O’Mallery’s [39] framework included:

*Stage 1*: Identifying the broad research question: What is known from the existing literature about immigrant and refugee health in Canada over the last two decades?

**Figure 1:**
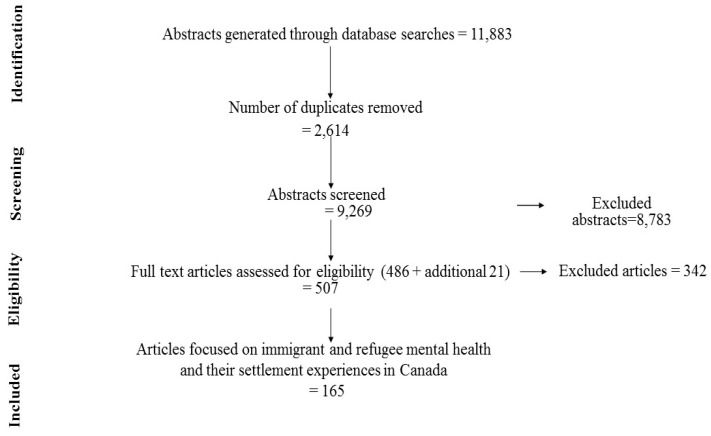
PRISMA flow diagram of review search screening process.

*Stage 2*: Identifying the relevant studies: For locating and identifying the articles, we conducted an electronic literature search in consultation with an experienced librarian for peer reviewed English language articles from January 1990-August 2013 in Medline, Embase, PsycInfo, Healthstar, ERIC and CINAHL databases which contained desired terms in the title, abstract or keywords. Furthermore, we searched the literature online databases for any relevant recent articles from September 2013–May 2015. The full set of search terms and inclusion and exclusion criteria for the selection of studies are given in Table 1. A flow chart for the search and selection and results are illustrated in Figure 1.

**Table 1:** Search Strategy & Selection.

Search Terms		
culture/cultural/multicultural AND/OR race/racial/racism AND/OR diversity/diverse AND/OR religious/religion/spirituality AND/OR ethnic/ethno/minority/ethno cultural AND/OR Health/Health beliefs/Mental health/Diseases/Chronic conditions AND immigrant/emigrant/migrant/immigration/refugee/newcomer/non-status/precarious AND Canada AND English		
**Inclusion Criteria**	**Exclusion Criteria**	**Resources Searched**
Written in English Focused on Canadian context Peer reviewed research articles Publication dates between 1990 and 2015 Primary and secondary research articles Articles best fit“ with the research question	Literature Reviews Gray literature Reports Thesis Dissertation Book Chapters Case Studies	Medline Embase PsycInfo HealthStar ERIC CINAHL

*Stage 3*: Selecting the studies: Due to the enormous number of studies on immigrant and refugee health we narrowed our focus to mental health of immigrants and refugees. Therefore, we modified our selection of studies to focus on immigrant and refugee mental health in Canada over the last two decades. We followed the WHO’s [3,40] conceptualization of mental health. After removing duplicates and irrelevant abstracts, 486 articles were chosen for full text review based on the above question and inclusion criteria. Full text of these 486 articles were exported electronically to Ref Works-COS, Pro Quest, LLC, a repository platform, to organize and store the references. A total of 165 articles [4,5,6,7,8,9,10,11,12,32,41,42,43,44,45,46,47,48,49,50,51,52,53,54,55,56,57,58,59,60,61,62,63,64,65,66,67,68,69,70,71,72,73,74,75,76,77,78,79,80,81,82,83,84,85,86,87,88,89,90,91,92,93,94,95,96,97,98,99,100,101,102,103,104,105,106,107,108,109,110,111,112,113,114,115,116,117,118,119,120,121,122,123,124,125,126,127,128,129,130,131,132,133,134,135,136,137,138,139,140,141,142,143,144,145,146,147,148,149,150,151,152,153,154,155,156,157,158,159,160,161,162,163,164,165,166,167,168,169,170,171,172,173,174,175,176,177,178,179,180,181,182,183,184,185,186,187,188,189,190,191,192,193,194,195] were selected for the final review.

*Stage 4*: Charting the data: These 165 articles were charted using Microsoft Excel to analyze in detail. Variables we charted include author/s, name of the journal, year of publication, title, aim of the study, focus area, study method and design, ethnicity, age, immigration status, gender, sample size, study setting, data collection, data analysis, major findings, limitations and implications for research, practice, and policy. Arksey and O’Malley’s [38] framework was useful in relation to our broad research question, and remained flexible to clarify concepts and to revise the research question as we became familiar with literature.

*Stage 5*: Collating, summarizing, and reporting the data: The findings from the study were analysed and collated into three broad themes.

## 3. Results and Discussion

### 3.1. Study Characteristics

Study characteristics are outlined in Table 2. All the studies were conducted in Canada, with almost half the articles reporting studies from province of Ontario (*n* = 72; 44%), followed by British Columbia (*n* = 28; 17%), Quebec (*n* = 24; 15%), Alberta (*n* = 20; 12%) and other provinces and multisite (more than one province) (*n* = 14: 8%). While a considerable number of the peer-reviewed published articles since 1990 focused on immigrants of all ethnicities, 34%of the articles relate to the experiences of South Asian immigrants. This is not a surprise considering that South Asians accounted for 25% of the total visible minority population and 4.8% of Canada’s total population [13].Study participants included South Asians, Chinese, Koreans, Filipinos, Arabs, Haitian, Vietnamese, Afro Caribbean, Europeans, and Hispanics. Some studies (20%) listed participants simply as “immigrants” and/or “refugees” without identifying the ethnic backgrounds of participants. Overall, 59% (*n* = 97) more than half of the studies used quantitative methodology, 37% (*n* = 61) used qualitative methodology, and remaining studies 4% (*n* = 7) followed mixed methods combining qualitative and quantitative methods. Almost all the studies 93% (*n* = 154) used cross sectional design and remaining 7% (*n* = 11) used longitudinal design. Twelve percent (*n* = 21) of the studies used data from national surveys to conduct the study. One shortcoming of the review appears to be that most of the studies follow cross sectional design and therefore results could not confirm whether relationships between variables are casual in nature. Participants in the studies were heterogeneous in nature as they arrived in Canada from diverse cultural and ethnic backgrounds and their definitions of health in general, mental health and settlement experiences differed. Additional limitation of the reviewed studies is the culturally diverse interpretations of survey questions [69,102]. Inclusion of multiple survey questions might have reduced misinterpretations of survey questions. As pre migration mental health issues might also influence mental health and service utilization in the settlement period, there is a need for more longitudinal studies to verify the association between length of residence in the host country and the mental health and wellbeing of ethnic minorities [4,42,196].

**Table 2:** Study characteristics.

Province Wide Distribution of Studies	N (*n* = 165)	%
Ontario	72	44
British Columbia	28	17
Quebec	24	15
Alberta	20	12
Multisite (more than one province)	14	8
New Found Land	3	2
Nova Scotia	1	<1
Manitoba	1	<1
Moncton	1	<1
Saskatoon	1	<1
Article type:		
Methods used: Qualitative	61	37
Methods used: Quantitative	97	59
Methods used: Mixed	7	4
Design:		
Cross sectional	154	93
Longitudinal	11	7

The findings of the scoping review revealed three important ways in which settlement is related to the mental health of immigrants and refugees: acculturative stress, economic uncertainty and ethnic discrimination.

### 3.2. Acculturative Stress

Acculturative stress refers to the difficulties immigrants face in relation to the process of adapting to the host society. Acculturations to western lifestyles hold significant consequences for the mental health of many diverse immigrant groups [175,197]. For example, acculturation to western food habits has been shown to produce negative health consequences such as chronic illnesses in ethnic groups such as Italians, eventually leading to depressive symptomology [175]. Weaker cultural orientation towards the host culture is also linked to more depressive symptomology especially in immigrant older adults [5,197]. Acculturation of parents/parental perception and emotional behavior of Chinese children at school (69, 65) showed that acculturation pressures thrust immigrants into an arena of competing identities. Immigrant and Canadian-born Chinese children had different experiences of social and psychological adjustment in the school. Among aspects of acculturation, English proficiency and participation in Chinese cultural activities were positively associated with social competence and negatively associated with adjustment problems, particularly in immigrant Chinese children. These results indicate the involvement of contextual factors in children’s social functioning and psychological well-being (73). The articles reviewed highlighted the impacts of post-migration acculturative-related stressors on the mental health of diverse immigrant groups [5,49,56,100,107,133,137,139,169,172,186,198] including Latin American men [140], immigrant mothers [111], and South Asian women [8,142].

Lack of social support and poverty that often accompany the migration and settlement process have been found to exacerbate mental ill health [52,63,80,102]. A part of the acculturation process is learning how to cope in Canada with limited social supports after immigration as migration disrupts many of the traditional supports that immigrants enjoyed in their home countries [123,141,157,165].

Immigrants might have less access to social supports and underutilize mental health services in Canada due to language difficulties, transportation issues and linguistically and culturally inappropriate services [32,48,57,60,69,75,78,83,86,96,98,132,134,138,176,199]. The positive relationship between social supports and mental health has been well established [4,44,46,51,52,53,54,55,56,57,58,59,79,200,201,202]. A supportive, protective and hospitable environment is also necessary for maintaining good health and mental well-being [67]. Neighborhoods disadvantage and lack of community involvement has been found related to health problems and illness in immigrant adults, children and youth [10,61,106]. Acculturative stress has been known to differ among refugee groups based on ethnicity, immigration status, gender, and generational status [45,64]. Ataca and Berry [158] examined the acculturation and adaptation of married Turkish immigrants and found that there were differences in the acculturation experience and adaptation of working class and professional immigrants. Gender differences were most apparent in the low socioeconomic group; women in general were more psychologically vulnerable than men [83,131]. Results provided evidence for the role of acculturation-related hassles or stressors in the psychological distress for Vietnamese Canadian students [172] and Somali refugees [100], Youth felt overwhelmed when trying to fit in with the new culture while maintaining components of their own [97]. Abouguendia and Noels [164] examined general and acculturation related daily hassles in first and second generation South Asians in Canada and suggest that the two groups have different acculturation experiences. Study on the predictors of psychological well-being of Pakistani immigrants in Toronto, Canada suggested that increased availability of social supports can moderate acculturative stress among Pakistani immigrants and their families [11].

Refugees who have undergone traumatic encounters might have reduced ability to cope with acculturation changes [100]. This does not mean, however that economic immigrants are necessarily spared from such trauma. Post-arrival stresses has been known to increase mental health risk for refugees [62,87]. Chinese sojourners were found to have experienced poorer psychological health after arrival than pre-departure [203]. While newcomers are potentially underserved category, the needs of settled immigrants are no less [8,204]. Long after migrating, Somali refugees, Ethiopian immigrants, first and second generation immigrant women *etc.* were found to be at risk for stress-related dysfunction, because they suffered from a diminished capacity to cope with acculturation challenges and exhibited this as somatic symptoms [50,100,124,205,206]. Emotional problems and learning difficulties have been found in refugee children [174,182]. Not all groups of migrants are negatively affected during the acculturation stage. For example, post traumatic adaptation and psychological health has been known to improve with departure from the conflict among war zone immigrants residing in Toronto [101].

Children with parents who have adapted well to Canada as well as maintained their traditional beliefs and practices tend to do better after migration than children whose parents have completely assimilated [207]. Proficiency in English as well as participation in cultural activities have been found to be positively associated with social competence and negatively associated with adjustment problems among immigrant children [73,178]. The literature informs us that those who immigrate to Canada in childhood (and therefore have greater English language proficiency have a higher risk of mental health challenges [106]. In addition, Islam *et al.*, [106] found that South Asian immigrants with better English/French proficiency had a higher risk of negative mental health outcomes.

### 3.3. Economic Disadvantage

The importance of financial resources for psychological and physical well-being of immigrant groups has been identified by a number of studies [6,32,33,74,171,184,208,209]. Many immigrants experience prolonged periods of low income and social exclusion in the post-migration context, which increase health disparities [34,210,211,212]. Research has pointed to the effects of poverty, un/under employment, financial insecurity and economic hardship on psychological health of immigrants at various stages of life [46,62,88,95,106,111,126,140,159,191,213,214], and of varied ethnicities [7,8,9,10,11,12,33,89,104,151,163,180,184].

Compared to native born persons, immigrants are more likely to be represented among the unemployed populations [215]. Unemployment can pose a mental health threat in three different ways [178]: it leads to poverty giving less opportunity to acquire education and access to quality health care; it is a frustrating and stressful experience that has the potential to lead to more mental health problems and illness; and, it leads to unhealthy coping strategies namely drinking, gambling, smoking or drug abuse [89]. Effects of unemployment may also vary based on generational status. For example, Zunzunegui and colleagues [129] studied the relationship between community unemployment and the health of first and second generation immigrants and found that among first-generation immigrants, community unemployment was associated with psychological distress.

There are contradictions in the research in relation to the effect of underemployment on immigrant mental health. Tang and colleagues [12] found that underemployed migrants or those who suffered occupational stress do not fare much better in terms of mental health compared to unemployed migrants. On the contrary a study on the Southeast Asian refugees by Beiser and colleagues [193] revealed that underemployment, which is a threat to the mental health of the permanent resident Canadians, did not jeopardize the mental health of refugees.

Lack of recognition of their international qualifications and skills is a barrier to immigrant employment in Canada [10,12]. Immigrants can feel depressed that their past education is irrelevant to their current work [9,126]. Economically disadvantaged individuals report diminished levels of self-esteem [216] and strained family relationships [149] and lower life satisfaction [42]. The most frequently mentioned rehabilitation goals by both Canadian-born and immigrant consumers in the psychiatric rehabilitation program that included immigrants pertained to improving consumers’ financial situation [136].

There are important intra-group differences in the relationship between economic disadvantage and migrant mental health. Beiser and colleagues [8] conducted a comparison of psychiatric illness in different cultures and demonstrated that though poverty created a risk of mental illness for both refugee and resident Canadians, the association between economic disadvantage and ill health proved stronger for refugees.

### 3.4. Ethnic Discrimination

Being a “visible minority” has been associated with high depressive symptoms as seen in the study of postnatal depression among immigrant women in Quebec [111]. Ethno-racial status and age emerged as key variables affecting the social exclusion of young immigrant mothers which in turn had deleterious effects on their health [105]. Research has found a relationship between perceived discrimination and psychological distress [156,178,195]. Depression symptoms, likely due to discrimination have also been found in samples of racialized immigrant university students and youths [76,193]. Whitley and Green [104] studied psychosocial experience of immigrant Black women in Montreal and reported three notable stressors emerged from their analyses: financial adversity, racism, and absent fathers. Furthermore, three notable buffers emerged from their analyses were families, the church, and cultural pride.

Discrimination has been identified as a social stressor [7,162,171,192,195]. Racial discrimination has been found to be an important risk factor for the mental health of diverse immigrant groups [66,84,104,115,121]. The results from a study by Noh and colleagues [121] emphasized the salience of subtle discrimination for the mental health of migrants. Beiser & Hou [7] revealed that when the Southeast Asians encountered racial discrimination or unemployment, ethnic identity attachment amplified the risk of depressive affect. By contrast, a strongly held ethnic identity provided a psychological advantage for individuals experiencing difficulties with the dominant language. Discrimination also influences service utilization. In a study of immigrants and refugees living with HIV/AIDS in Toronto, Chen and colleagues [41] found that when participants encountered discrimination from medical practitioners it added to their stress and discouraged them from utilizing support in the future.

In summary, acculturation related stressors including the loss of social support in the migration process can impact mental health. Acculturative stress differs among immigrant groups based on ethnicity, immigration status, gender, and generational status. Immigrant mental health is impacted by their negative employment experiences in the settlement period and resultant economic hardships. Ethnic discrimination can also contribute to depression and psychological distress. It can also lead to barriers in the utilization of mental health services.

### 3.5. Recommendations

This scoping review provides insights into important areas for public health interventions. Public Health practitioners need to be cognizant of the impacts of settlement and its related features- acculturative stress, economic difficulties and the experiences of discrimination while designing their interventions. There is a need to recognize the multiple and intersecting oppressions faced by immigrants and the community context in which they live [97]. Such an orientation recognizes the ways in which race, class, gender, age, sexual orientation and newcomer status work simultaneously to marginalize immigrant groups. The findings of the review call for health practitioners to recognize the vulnerability of immigrants in the settlement process and provide immigrants with resources to improve and expand their social networks. It reaffirms the importance of incorporating Social Determinants of Health into a holistic health promotion intervention and to advocate for more effective policies to facilitate newcomer settlement.

Increased availability of social supports can moderate acculturative stress [11,102,197,217]. Health practitioners need to help immigrants improve and access social and professional networks through programs such as mentorship or host programs. School-based interventions would be effective to reach out to immigrant parents [62,94,218]. A positive relationship between parents and schools offers opportunities to strengthen social supports that help parents adapt and in turn promote healthy emotional behavior in children [65,185].

A health promotion approach can be a useful one in approaching issues of immigrant mental health [97,219]. Such an approach can contribute toward addressing the social determinants of mental wellbeing and reduce health disparities for diverse immigrants [11,92,106]. Effective mental health promotion must consider the social determinants of health, and integrate the principles of social inclusion, access and equity into practice [79,90,220]. Ideally, mental health education would be integrated into the supports and services currently available to immigrants and their families [81]. Culturally appropriate public education and media campaigns should be developed and targeted to specific communities, using imagery and messages those are acceptable to community members. In order to improve mental health service, accessibility and delivery has to be re-examined considering community values, strengths, weaknesses and social inequities that help or hinder mental health promotion, and access to appropriate services for ethno linguistic communities enhanced through new models of collaborative care and services [122,137]. Early intervention services should monitor the pathways to care for young people of diverse ethnic backgrounds living within increasingly multiethnic and multilinguistic societies to address any disparities in accessing care [70].

The 2007 Mental Health Strategy for Canada ([221], p. 60) recognizes that immigrants and refugees face specific challenges that “puts their mental health at higher risk” including employment challenges and barriers to help seeking. The strategy recommends linguistically and culturally sensitive practice with immigrants groups. The strategy also recommends the development and implementation of mental health plans that meets the needs of immigrants and newcomers with their involvement ([221], p. 62). The authors of this paper suggest that such a plan should be broadened to also include strategies that promote immigrants’ economic and social inclusion into society.

Health practitioners have an important role to play in advocating for better public policies impacting the settlement of newcomers and their mental health. Policy makers need to recognize the systemic barriers encountered by foreign-trained professional immigrants and ensure mechanisms for the fair evaluation of immigrant credentials [12]. Research has pointed out that limited policies exist to support the health of multicultural populations [7,12,61,84,85,92,151]. The Canadian health care system is one of the best in the world, yet one sees the contradictions that reveal the deeply structured hierarchies based on race, class and gender [122]. There is also a need for systemic change to formulate collaborative, community-based strategies for mental health promotion and interventions [84,90].

There is a need for more longitudinal research exploring the relationship between immigrant settlement and mental health. Ethnographies of single cultural groups, while often rich in depth and detail, do not examine shared adaptation experiences across diverse ethnic communities, nor can they readily inform policy and program in healthcare system that are required to serve multicultural client population [84].

Research has also identified the need to change social and professional attitude towards culturally and diverse individuals, and towards people who are economically disadvantaged [213,222,223,224], and who need culturally competent care [170,175]. There is also a clear need for mental health practitioners from diverse ethnic and linguistic backgrounds [149,223,224].

## 4. Conclusions

The findings from the scoping review identified that settlement related experiences are important to consider in relation to immigrant mental health. The findings call for public health practitioners to be cognizant of these relationships in their interventions. There is a need for practitioners to intervene at the preventive level to address settlement related stressors and to advocate for policies that will effectively address labour market inequalities and discrimination in society.

The results of this scoping review are compatible for what has been described for other countries like USA, Australia, France and Germany. Immigration to Canada, USA, Australia, France and Germany accounts for about 93% of total migratory flows globally. Among the four countries, Canada and Australia receive the highest number of immigrants in the world [225,226]. Although immigration policies in Canada, Australia are similar, it is slightly different for USA and UK. All these countries have English as an official language. Canada and UK provide free healthcare. The experiences/difficulties faced by immigrants during the settlement phase in their host countries and their impact on mental health and well being are compatible. For e.g., Changes that occur in immigrants’ employment structure are common in Canada, USA, UK and Australia and these changes can have a significant effect on psychological well being and adaptation [180,226].

### Limitations

A limitation of the study is that we did not conduct bibliographic and/or grey literature searches. Furthermore, we did not assess the quality of the studies nor the methods used by each researcher. We reviewed articles written and published in English only. This might have led to omission of interesting research published in other languages especially French given the focus on Canada. Another short coming of this review is that we did not differentiate between immigrants and refugees. Although we knew that the pre-and post –migration experiences and mental health well-being/issues can be different for immigrants and refugees, there was no possibility for us to undertake two different scoping reviews for the two groups with the available time and resources. Another limitation to note is that since most of the studies followed cross sectional design, relationships between variables may be casual in nature and this may affect the results.
